# Anomaly recognition in surveillance based on feature optimizer using deep learning

**DOI:** 10.1371/journal.pone.0313692

**Published:** 2025-05-19

**Authors:** Shaista Khanam, Muhammad Sharif, Mudassar Raza, Waqar Ishaq, Muhammad Fayyaz, Seifedine Kadry

**Affiliations:** 1 Department of Computer Science, COMSATS University Islamabad, Wah Campus, Wah Cantt, Punjab, Pakistan; 2 Department of Computer Science, Namal University, Mianwali, Pakistan; 3 Telecommunication Department, Hazara University, Mansehra, Pakistan; 4 Department of Computer Science, FAST - National University of Computer and Emerging Sciences, Chiniot-Faisalabad Campus, Chiniot, Pakistan; 5 Department of Computer Science and Mathematics, Lebanese American University, Beirut, Lebanon; 6 Department of Applied Data Science, Noroff University College, Kristiansand, Norway; King Khalid University, SAUDI ARABIA

## Abstract

Surveillance systems are integral to ensuring public safety by detecting unusual incidents, yet existing methods often struggle with accuracy and robustness. This study introduces an advanced framework for anomaly recognition in surveillance, leveraging deep learning to address these challenges and achieve significant improvements over current techniques. The framework begins with preprocessing input images using histogram equalization to enhance feature visibility. It then employs two DCNNs for feature extraction: a novel 63-layer CNN, “Up-to-the-Minute-Net,” and the established Inception-Resnet-v2. The features extracted by both models are fused and optimized through two sophisticated feature selection techniques: Dragonfly and Genetic Algorithm (GA). The optimization process involves rigorous experimentation with 5- and 10-fold cross-validation to evaluate performance across various feature sets. The proposed approach achieves an unprecedented 99.9% accuracy in 5-fold cross-validation using the GA optimizer with 2500 selected features, demonstrating a substantial leap in accuracy compared to existing methods. This study’s contribution lies in its innovative combination of deep learning models and advanced feature optimization techniques, setting a new benchmark in the field of anomaly recognition for surveillance systems and showcasing the potential for practical real-world applications.

## Introduction

Surveillance is described as observing, tracking, documenting, and controlling the behavior of individuals, objects, and events to regulate activity in today’s social life [1]. To be safe and secure is one of the basic human needs [2]. The unexpected data patterns cause some problems that are referred to as anomalies. However, due to the wide variety of specific contexts, unusual event detection is a difficult glitch to solve [3]. Theft, robbery, shoplifting, snatching, automobile burglary, arson, and running arbitrarily are all examples of security threats [4,5]. These security threats can be minimized by implementing modern techniques [6,7] of computer vision [8] and Deep Learning. Identifying anomalous frames and categorizing suspicious photos is complicated and time-consuming [9]. Numerous applications use anomaly recognition, such as intrusion detection applications, visual monitoring, and recognition of suspicious or anomalous behavior. Surveillance is usually done by different types of cameras, all public and private places need proper surveillance [10].

Detection of anomalous patterns in surveillance [11] is much more challenging and complex now because of massive and instantaneous data handling requirements. Human beings have some limitations. They cannot examine enormous data for anomaly recognition in surveillance scenes [12]. Prior automated warning of violent activity in surveillance scenes could significantly minimize the risk of any dangerous activity. Anomaly incident recognition, a well-known research part, is a sub-classification of insightful reconnaissance using computer vision techniques. The outcomes of this classification can be used in various applications, including avoiding security threats in public places and other routine surveillance at various locations[10].

In today’s smart city era, video monitoring has become extremely vital. Large surveillance cameras are installed in public and reserved locations to monitor infrastructure and public safety. To separate the anomalous frame from normal footage is much time and effort, consuming [13]. The categorization of suspicious photos is complicated by variations in shape, texture, people orientation, size, and background. Anomalous image classification is also discovered as an important issue in the domain of surveillance[14]. Some techniques and algorithms are utilized under the umbrella of indirect supervision for anomaly recognition [15].

This paper addresses issues of accuracy, robustness, and the shortcomings of previous approaches by presenting a unique framework for anomaly identification in surveillance systems. The following are this work’s main contributions:

1. Introduction of Up-to-the-Minute-Net: This research proposes a new 63-layer DCNN named Up-to-the-Minute-Net, specifically designed for feature extraction in anomaly detection tasks. This model is optimized to handle complex image data, enhancing its effectiveness in identifying suspicious activities in surveillance footage.
2. Dual Feature Extraction Mechanism: This approach combines the feature extraction capabilities of the proposed Up-to-the-Minute-Net with the well-established Inception-Resnet-v2 model. By fusing features from both networks, we create a rich and diverse set of features that improve the model’s ability to detect subtle anomalies.
3. Advanced Feature Selection Techniques: To further refine the feature set, we introduce two state-of-the-art optimization algorithms—Dragonfly and GA—for feature selection. These methods ensure that only the most relevant features are used, significantly enhancing the model’s performance and reducing computational complexity.
4. Extensive Cross-Validation: We rigorously evaluate our model using 5-fold and 10-fold cross-validation, demonstrating its robustness and generalizability. The proposed framework achieves an unprecedented 99.9% accuracy through these experiments, substantially improving the existing technique.
5. Benchmark Dataset and Real-World Application Potential: The model is tested on the Suspicious Activity Recognition (SAR) benchmark dataset, and its performance surpasses existing state-of-the-art methods. Our model demonstrates strong potential for practical, real-world applications, particularly enhancing public safety through more accurate and reliable surveillance systems.

The manuscript has six sections. Section 1 is about the introduction of the topic. Section 2 belongs to similar jobs. Section 3 encircles the proposed deep neural network-based methodology with the feature optimizer used in the introduced process. Section 4 is about the experimental outcomes and discussion, Section 5 is about discussion and limitations, and Section 6 depicts the conclusion and future direction.

### Nomenclature used in the paper

Several methods are discussed in this review paper, and a list of nomenclature is also provided in Table 1, which are used throughout the paper.

**Table 1:** A list of abbreviations used in this paper.

Nomenclature	Referred to	Nomenclature	Referred to
CNN	Convolutional Neural Network	FC	Fully Connected
DCNN	Deep Convolutional Neural Network	FMS	Feature Map Size
YOLO	You Only Look Once	FD	Filter Depth
ILR	Initial learning rate	ST	Stride
MB	Mini batch	PD	Padding
MV	Momentum Value	CN	Convolutional Layer
SVM	Support Vector Machine	Quad-SVM	Quadratic SVM
FineG-SVM	Fine Gaussian SVM	CoarG-SVM	Coarse Gaussian SVM
MedG-SVM	Medium Gaussian SVM	TP	True Positive
FP	False Positives	TN	True Negatives
FN	False Negatives	GA	Genetic Algorithm
F1-Sc	F1-Score	AUC	Area Under the Curve
D	Dropout	BN	Batch Normalization
P	Pooling	ADD	Addition
GCN	Group Convolutional	CL	Classification
Cu-SVM	Cubic-SVM	R	Relu
CM	Confusion Matrix	MB	Mini Batch

## Related work

Any recognition system mainly follows the important steps 1. pre-processing [16], 2. feature extraction[17,18] 3. optimal feature selection [19,20], and 4. classification [21,22] notwithstanding their complexities and complications. Before being used in the construction and application of models, images must undergo image preprocessing [23]. This includes, but is not limited to, scaling, organizing, and color modifications. Scale Invariant Feature Transform(3DSIFT) [24] finds the nearby elements in a picture. A one-dimensional CL (CONVID) is employed to limit commotion and to get applicable data from the embedding produced by the spatial highlights’ extractor [25]. A deep neural network gives very exceptional outcomes in the area of surveillance for anomaly detection, as the methodology proposed by experts is to perceive and segment individuals in pictures and recordings [26–28]. Features are extracted from the images of suspicious actions and some methods such as MPPCA [29], MMPC+SFA [30], Conv-AE [31], ConvLSTM-AE [32], Deep Generic [33], GANs [34] are proposed for feature extraction. Heuristic and hand-crafted features a 3D extension of HOG [35] and HOF [36] provide a brilliant outcome. These features are also assessed utilizing diverse deep-learning models. Some pre-trained feature extraction models such as VGG -19 [37], Inception -3 [38], ResNet-50 [39], Google Net [40], ResNet-18 [41], Squeeze Net [42] DL strategies are utilized for feature extraction. Further with the features selection techniques, knowledge can be discovered and mined which allows for the elimination of redundant and outdated features while maintaining the core details [43].

Some irrelevant features can be further eliminated using dimension-reduction methods to improve the system’s effectiveness and performance [44–46]. Principal Component Analysis(PCA) [47] attempts to order all n information vectors as immediate groupings with few eigenvectors while ignoring test focuses that don’t match the standards [48]. Object identification [49], object tracking [50], and object recognition [51] are the three phases of surveillance. Object classification would ultimately allow for distinction not only between humans and vehicles [52] but also between various types of behavior, such as drunk drivers and suspicious humans [52]. Members of the intoxicated driver’s class are likely to trigger an accident, so they are indirectly conducting behavior prediction. Members of the suspicious human class who are discovered in a parking lot may attempt to steal a car [53].

In the domain of surveillance, walker grouping is a significant objective recognition concern [54]. DL has recently seen a considerable advancement in images such as object recognition, and pose estimation, as different tasks utilizing non-direct relations in high-dimensional information [55,56]. In Generative Adversarial Networks (GAN), in light of its capacity to learn successive information, CNN-based LSTM [57,58] is utilized for encoding transient data in pictures [59]. Some methods are used for object detection which leads to object classification as YOLO is proposed for object detection [60]. Table 2 presents a comparative comparison of the state-of-the-art deep learning approaches for anomaly identification in surveillance.

**Table 2:** Comparative analysis of existing approaches in anomaly recognition for surveillance systems.

Study	Year	Techniques Used	Performance Metrics	Data Type/ Context	Strengths	Limitations
[61]	2024	Transformer Blocks, Margin Learning, Hard Score Memory, Pseudo-Labels for Anomalous Events	Accuracy, AUC, Pre, Rec, and F1-Sc	Video Data Shanghai Tech Dataset UCF-Crime Dataset	Temporal Continuity, Improved Detection, Discriminative Power, Competitive Performance	Computational Complexity, Dependence on Pseudo-Labels, Potential Overfitting, Interpretability Challenges
[62]	2023	Transfer Learning, Model Fusion, Multi-task Classification	97.99% Accuracy	RLVS Dataset	First to solve the generalization issue in video anomaly detection	The unseen dataset accuracy (87.25%) is slightly lower, indicating potential for improvement on entirely new data.
			83.59% Accuracy	UCF Dataset		
			87.25%	Unseen Dataset		
[63]	2023	Collaborative Learning, Unsupervised Video Anomaly Detection, Server Knowledge Accumulation, Pseudo-Label Refinement, FedAVG	AUC Baseline: 76.2% With SKA: 77.1%, With SKA + PLR: 78.02%, Ablation Study	UCF-Crime Dataset, XD-Violence Dataset	Fully unsupervised, Scalable to multiple participants, and Effective at localizing anomalous events without labeled data.	Performance drops with a higher number of participants due to reduced data per participant.
[64]	2023	Deep Learning Models, Time Series Forecasting and Anomaly Detection, Statistical Feature Selection, Preprocessing Techniques, Ensemble Learning	Accuracy, Numenta Anomaly Benchmark (NAB), Credit Card Fraud Detection, Pre/Rec/F1-Sc	Multidimensional Time Series Data, Credit Card Fraud Dataset	Effective High-Dimensional Feature Selection, Deep Learning Models Capture Complex Patterns, Wide Application in Various Fields, Improved System Performance with Ensemble Models	Challenges with LSTM Training, Sensitivity to High-Dimensional Data, Limited Generalization across Different Domains
[65]	2024	Streaming Architecture with Apache Kafka Random Forest Algorithm, Threshold-Value Based Point Anomaly Detection, Publish-Subscribe Model	Accuracy-anomaly detection accuracy was evaluated, achieving 96.7% in a single-node setup and 98.6% in a distributed-node setup, Computation Time- showing results 37.5 ms in single-node and 38.5 ms in distributed-node configurations, Data Miss- Evaluated for data processing efficiency, ensuring no data loss during stream ingestion and anomaly detection.	Streaming Data, Case Study	High Accuracy in Real, Efficient for Large-Scale, High-Speed Data Streams, Low Computation Time, Scalability	No Mention of Specific Anomaly Types, Kafka Dependency, or Potential Overhead in Distributed Setup
[66]	2024	Xception ResNet-50 AlexNet VGG-19, Transfer Learning	Accuracy (on test data): Transfer Learning Xception: **99.92%** (small wind turbine dataset) and **100%** (large-scale turbine dataset) VGG-19: **99.83%** Xception (base): **99.33%** ResNet-50: **93.27%** Custom CNN: **80.77%**	6000 RGB images Large-scale wind turbine blade	- High Accuracy in Anomaly Detection - Effective Handling of Small Datasets - Generalization Across Different Turbine Sizes - Scalability of the Architecture - Practicality Through Autonomous Drone Data Collection	- Limited to Binary Classification - Small Dataset Size for Large-Scale Turbines - Focus on Stationary Turbines, Causing Energy Production Losses - Lack of Fault Localization and Size Estimation - Blurring of Images for Rotating Blades During Inspection

### Motivation

The proposed research is motivated by the critical need to enhance surveillance systems to ensure public safety in an increasingly complex environment where manual monitoring is no longer feasible. Current DL methods for anomaly recognition face challenges in achieving high accuracy, efficient feature extraction, and scalability for real-world applications. To bridge these gaps, this paper introduces a novel framework that combines two DCNNs— the custom-built “Up-to-the-Minute-Net” and the established Inception-ResNet-v2—for comprehensive feature extraction. This dual approach is complemented by a hybrid optimization technique using Dragonfly and GA for feature selection. This approach notably enhances the model’s performance by concentrating on the most pertinent features. The model’s ability to achieve 99.9% accuracy with 5-fold cross-validation demonstrates its superiority over existing methods. By advancing the state-of-the-art in both feature extraction and selection, this study advances not only the theoretical knowledge of anomaly recognition but also offers a practical solution that is scalable and applicable to real-world surveillance scenarios.

## Proposed methodology

**Fig 1:**
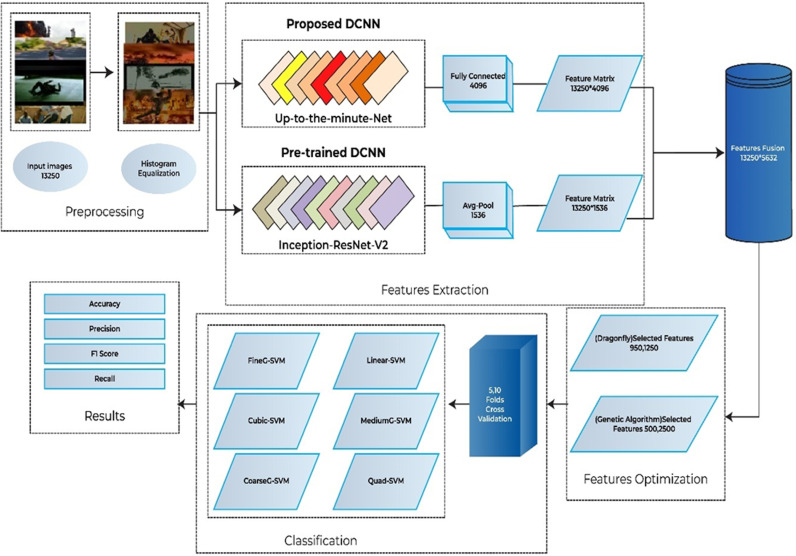
Flowchart of the proposed approach.

The main objective of this study is to introduce a method for detecting anomalies in surveillance footage and identifying suspicious activities. The overall architecture of the proposed anomaly detection framework is illustrated in (Fig 1). It shows the integration of various stages, including the initial image preprocessing step using contrast enhancement [67] and histogram equalization. In this manuscript, a DDCNN named Up-to-the-Minute-Net is proposed. The other significant phases performed are feature extraction from the proposed network and pre-trained deep network Inception-Resnet-V2 [68]. This visualization helps in understanding how each component contributes to the overall anomaly detection process, from preprocessing to feature extraction and classification.

Extracted features from both networks are fused in the fusion phase. The extracted features were passed to two feature optimizers Dragonfly and GA. The methodology produces improved outcomes as compared to much other research after the classification. The optimized features are transferred to distinctive classifiers for categorization. The experiments are performed on two different cross-validation numbers, 5- and 10 values are selected for this purpose.

### Data preprocessing

Before processing the images through the networks, the data undergoes several preprocessing steps. Initially, contrast enhancement [67] and histogram equalization techniques are applied to improve image quality. The noise from the image is eliminated using a contrast enhancement technique and histogram equalization upgrades the differentiation of pictures by changing the qualities in the intensities of the image. Histogram equalization further improves the contrast by redistributing the image’s intensity levels, making features more distinguishable.

### Feature extraction

Feature extraction is integral to the execution of the classification procedure. The research has a prodigious contribution to the fusion of features extracted from the proposed Up-to-the-minute-Net and pre-trained deep Inception-Resnet-V2 [68] network. Both networks are used for feature extraction from the SAR [69] dataset. The features extracted from Up-to-the-Minute-Net are 4096 and features extracted from Inception-Resnet-V2 are 1536.

#### Proposed Deep CNN Up-to-the-Minute-Net.

A new CNN-based deep neural network named Up-to-the-Minute-Net is proposed. First, the CIFAR-100 is used to train the anticipated CNN model [70]. The dataset contains 100 classes of images belonging to different categories. In the wake of switching the proposed network over to a pre-trained model, the features are extracted from the SAR [69]. The proposed Up-to-the-Minute-Net is a branched network. Table 3 elaborates on the features FMS, FD, ST, PD, and the pooling window size of each layer.

**Table 3:** Detailed layers’ configuration of proposed Up-to-the-Minute-Net.

Layer	Layer Name	FMS	FD	ST	PD	Pooling Window Size
1	Input	227 × 227 × 3	11 × 11 × 3 × 96	–	–	–
2	CN1	55 × 55 × 96	–	[4,4]	[0,0,0,0]	–
3	R1	55 × 55 × 96	–	–	–	–
4	CN2	55 × 55 × 48	1 × 1 × 96 × 48	[1,1]	Same	–
5	BN1	55 × 55 × 48		–	–	1 × 1 × 48
6	CN3	55 × 55 × 96	11 × 11 × 48 × 96	[1,1]	Same	–
7	LR1	55 × 55 × 96	–	–	–	Scale 0.01
8	CN4	55 × 55 × 96	5 × 5 × 96 × 96	[1,1]	Same	–
9	BN2	55 × 55 × 96		–	–	1 × 1 × 96
10	LR2	55 × 55 × 96		–	–	–
11	BN3	55 × 55 × 96		–	–	–
12	ADD1	55 × 55 × 96		–	–	–
13	CN5	55 × 55 × 96	1 × 1 × 96 × 48	[1,1]	Same	–
14	BN4	55 × 55 × 96	–	–	–	1 × 1 × 48
15	CN6	55 × 55 × 96	11 × 11 × 48 × 96	[1,1]	Same	–
16	LR3	55 × 55 × 96	–	–	–	Scale 0.01
17	CN7	55 × 55 × 96	5 × 5 × 96 × 96	[1,1]	Same	–
18	BN5	55 × 55 × 96	–	–	–	1 × 1 × 96
19	LR4	55 × 55 × 96	–	–	–	Scale 0.01
20	BN6	55 × 55 × 96	–	–	–	1 × 1 × 96
21	ADD2	55 × 55 × 96	–	–	–	–
22	N1	55 × 55 × 96	–	–	–	–
23	P1	27 × 27 × 96	–	[2,2]	[0000]	Max pool 3 × 3
24	BN7	27 × 27 × 96	–	–	–	1 × 1 × 96
25	GC8	27 × 27 × 256	5 × 5 × 48 × 128 × 2	[1,1]	[2222]	–
26	R2	27 × 27 × 256	–	–	–	–
27	N2	27 × 27 × 256	–	–	–	–
28	P2	13 × 13 × 256	–	[2,2]	[0000]	–
29	BN8	13 × 13 × 256	–	–	–	1 × 1 × 256
30	GC9	13 × 13 × 384	3 × 3 × 256 × 384	[1,1]	[11]	–
31	R3	13 × 13 × 384		–	–	–
32	CN10	13 × 13 × 192	1 × 1 × 384 × 192	[1,1]	Same	–
33	BN9	13 × 13 × 192	–	–	–	1 × 1 × 96
34	CN11	13 × 13 × 384	5 × 5 × 192 × 384	[1,1]	Same	–
35	LR5	13 × 13 × 384	–	–	–	–
36	CN12	13 × 13 × 384	3 × 3 × 384 × 384	[1,1]	Same	–
37	BN10	13 × 13 × 384	–	–	–	–
38	LR6	13 × 13 × 384	–	–	–	–
39	BN11	13 × 13 × 384	–	–	–	–
40	ADD2–1	13 × 13 × 384	–	–	–	–
41	CN13	13 × 13 × 192	1 × 1 × 384 × 192	[1,1]	Same	–
42	BN12	13 × 13 × 192		–	–	–
43	CN14	13 × 13 × 384	5 × 5 × 192 × 384×	[1,1]	Same	–
44	LR7	13 × 13 × 384	–	–	–	–
45	CN15	13 × 13 × 384	3 × 3 × 384 × 384	[1,1]	Same	–
46	BN13	13 × 13 × 384	–	–	–	1 × 1 × 384
47	LR8	13 × 13 × 384	–	–	–	–
48	ADD2–2	13 × 13 × 384	–	–	–	–
49	GC16	13 × 13 × 384	3 × 3 × 192 × 192 × 2	[1,1]	Same	–
50	R4	13 × 13 × 384	–	–	–	–
51	GC17	13 × 13 × 256	13 × 13 × 192 × 182 × 2	[1,1]	Same	–
52	R5	13 × 13 × 256	–	–	–	–
53	P5	6 × 6 × 256	–	–	–	Max pool 3 × 3
54	BN14	6 × 6 × 256	–	–	–	1 × 1 × 384
55	FC18	1 × 1 × 4096	–	–	–	–
56	R6	1 × 1 × 4096	–	–	–	–
57	D6	1 × 1 × 4096	–	–	–	Dropout 50%
58	FC19	1 × 1 × 4096	–	–	–	–
59	R7	1 × 1 × 4096	–	–	–	–
60	D7	1 × 1 × 4096	–	–	–	Dropout 50%
61	FC20	1 × 1 × 4096	–	–	–	–
62	SOFTMAX	1 × 1 × 4096	–	–	–	–
63	CL	–	–	–	–	–

**Fig 2:**
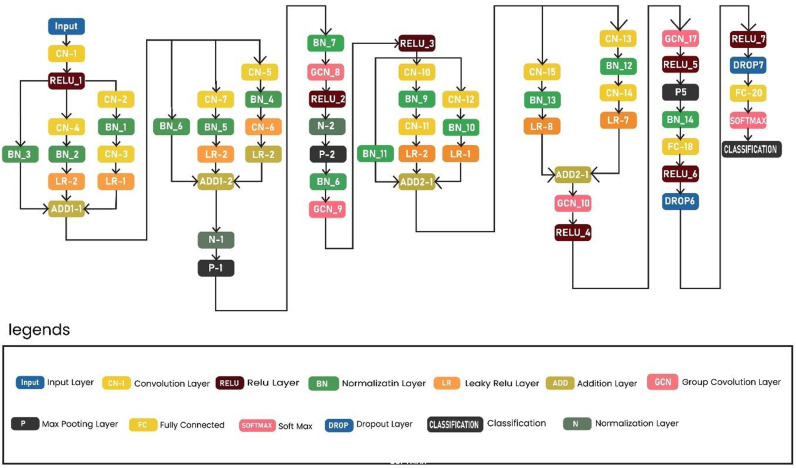
Detailed layered architecture of proposed Up-to-the-Minute-Net.

The proposed deep CNN net starts from the Input layer. Its FMS is 227 × 227 × 3 and the FD is 11 × 11 × 3 × 96. Following the input, the network has a CN1 which CN layer. The network has a total of 63 layers in number. The features are extracted from FC20 in the form of 1 × 1 × 4096. The detailed architecture is depicted in (Fig 2). The network has 20 CN layers. It also contains 7 R layers, 2 D layers, 3 FC layers, and one SoftMax layer. The network also has 14 BN layers, 5 P layers, 4 ADD layers, 4 GCN layers, and 1 final CL layer for classification purposes. The proposed deep CNN net starts from the Input layer. Its FMS is 227 × 227 × 3 and the FD is 11 × 11 × 3 × 96.

The network output type is classification. Some parameters are definite and have to be set at the time of implementation. Table 4 describes the hyperparameters and their values. These hyperparameters have a major role in the training of the network.

**Table 4:** Hyperparameters and their values for proposed Up-to-the-Minute-Net.

Hyperparameter	Value
Epochs	30
ILR	0.001
Optimization	Stochastic gradient descent with momentum
MB	128
MV	0.9

All dataset images contain features that were obtained from the FC20 layer. Each picture has 4096 total features that were obtained from this layer. The feature matrix’s overall size thus increases to 13250 by 4096. The first network used for feature extraction is the proposed Up-to-the-Minute-Net.

#### Inception-Resnet-V2.

The second network used for feature extraction is Inception-Resnet-V2 [68]. This DCNN is trained on more than 1,000,000 images from the ImageNet [71] data set without the FC layer. The net is 164 layers deep and can categorize images into 1000 article classes. Thus, the network has acquired fine feature portrayals for a large number of images. The network has an image input size of 299 by 299. In the Inception-Resnet block, numerous valued CN channels are joined with available connections. Inception-Resnet-V2 has a total of 824 layers and functions in number. Its input type is the image, and its output type is classification. The features are extracted from the average P-layer. The total features extracted from the network are 1536 and the total images in the SAR [69] are 13250. The feature matrix we achieved is 13250* 1536.

### Feature fusion

Feature fusion intends to consolidate the source image of a similar scene to shape one composite picture that holds a more exact depiction of the view than any of the singular source images. Preceding the merging of images, striking features, present in all source pictures, are separated utilizing a suitable component extraction method. The research has a prodigious contribution to the fusion of features obtained from the proposed Up-to-the-Minute-Net and pre-trained deep CNN network Inception-Resnet-V2. Both networks are used for feature extraction. Then, at that point, fusion is performed utilizing the features extracted from these networks. The extracted features from InceptionResNet-v2 are 1536 and Up-to-the-Minute-Net are 4096. These features got fused and made a collective compound of 13250*5632 fused features. The remarkable features are first recognized in each source image. The remarkable quality of a component is figured as a coefficient. The features fusion process ensures that the majority of the predominant features are integrated.

### Feature optimization

There is an immense number of retrieved features in the feature extraction phase. After the fusion process, this makes a gigantic combination of 5632 features. The training phase for classification can be slowed down for these many features. Additionally, concerns like indefinite features and the curse of dimensionality can emerge, thus, a decline in execution can happen. To resolve this issue, the fused features are delivered to the Dragonfly [72] and GA [73] feature optimizers simultaneously. These algorithms were chosen because of their demonstrated effectiveness in solving high-dimensional optimization problems, which is essential in the context of anomaly recognition and feature selection from large datasets.

#### Dragonfly algorithm.

The mathematical implementation of Dragonfly is as follows according to Reynolds, the overall behavior follows three primitive principles of separation, alignment, and cohesion [74]. While calculating the separation m is the position of the current individual,$\;m_{k}$ shows the position of the kth neighboring individual and n is some neighboring individuals.


$$S_{a}=-\sum_{k=1}^{n}m-m_{k}$$ (1)

While calculating alignment $w_{k}$ shows the velocity of the kth neighboring individual.


$$A_{a}=\frac{\sum_{k=1}^{n}w_{k}}{n}$$ (2)

The cohesion is calculated as follows:


$$c_{a}=\frac{\sum_{k=1}^{n}m_{k}}{n}-m$$ (3)

Where m is the position of the current individual [72]. These principles are incorporated into the Dragonfly Algorithm to guide the search process toward optimal solutions in a high-dimensional space.

#### Genetic algorithm.

GA reached to optimal solution through the probabilities of crossover and mutation by continuously changing the search space. In the following equation e is the number of generations and E is the total number of evolutionary generations set by the population [75].


$$R=\left(E+2\sqrt{𝕖}\right)/3E$$

While performing crossover two parents are chosen for mating one parent donates some part of genetic material and the corresponding part of the other parent participates in the offspring [76].

Dragonfly and GA were chosen due to their demonstrated suitability for feature selection in complex, high-dimensional datasets. Both algorithms are well-suited to balancing exploration and exploitation, which is critical for ensuring that the most relevant features are selected from the fused feature set. The number of optimal features selected from the Dragonfly features optimizer is 950 and 1250 and the total of optimal features chosen from the GA optimizer are 500 and 2500.

### Classification

The chosen features are pushed ahead to different classifiers. The picked classifiers are the variants of SVM [77]. The variants of SVM incorporate Quad-SVM [78], Linear-SVM [79], FineG-SVM [80], CoarG-SVM), MedG-SVM, and Cu-SVM [81]. The SVM classifier and its kernel can be found in [82–84]. The classifiers are evaluated on several performance evaluation metrics. The itemized upshots and trials are presented in the results section. The results are taken through two different folds of cross-validation numbers with two changed feature optimizers Dragonfly and GA.

## Experimental results and discussion

A total of 8 experiments are performed, the number of experiments performed with the Dragonfly is 4, and likewise, the experiments performed with GA are 4 in number. The dataset used for implementation purposes is SAR [69]. The features are extracted from two DCNNs, one is proposed Up-to-the-minute-Net, and the second is pre-trained Inception-Resnet-V2. The extracted features from both DCNNs got fused and provided a combination of 5632. Results are taken by 5- and 10-fold cross-validation. The number of optimal features selected is 500, 950, 1250, and 2500. The total number of iterations is 25 for each experiment. The presented research is implemented using MATLAB R2021a and on the Microsoft Windows 10 Pro operating system. The system has the processor of Intel(R) Core (TM) i5-2520M CPU @ 2.50GHz, 2501 Mhz, 2 Core(s), 4 Logical Processor(s), and BIOS Version Hewlett-Packard 68SCF Ver. F.67. The system has Installed Physical Memory (RAM) of 8.00 GB. The detailed results of all experiments are described in section 4.2. The performance measures used for experiments are Precision(Pre) [85], Recall(Rec) [86], Accuracy [87], F1-Sc [88], ROC, and AUC [89]. The performance criteria are similar for all experiments.

### Dataset used for implementation

A dataset containing five anomaly classes is arranged by acquiring four classes ((a)Falling (b)Fighting (c)Firing and (d) Running) from HMDB51 (https://www.kaggle.com/datasets/easonlll/hmdb51) [79] and one class (Fire) from AIDER (https://zenodo.org/records/3888300#.XvCPQUUzaUk) [80] datasets. The dataset is named SAR (Suspicious Action Recognition). It is also used in [59].

### Simulation and data details

**Fig 3:**
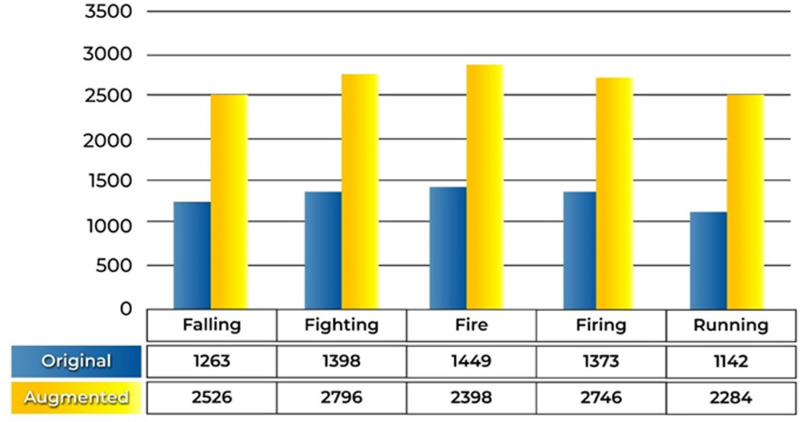
Graphical representation of original and augmented images.

This dataset SAR is essential for assessing how well the suggested anomaly recognition methodology works. The SAR dataset comprises 13,250 images, each with different scenarios and conditions, making it suitable for testing the robustness and accuracy of the proposed models. The total number of original images in the dataset is 6625 and the augmented images are 13250. The dataset distribution between original and augmented images is depicted in (Fig 3), where the blue bars represent the original images, and the yellow bars indicate the augmented images. The figure shows how the dataset has been expanded through augmentation to create a larger, more diverse dataset that improves the model’s training and reduces overfitting.

The combination we obtained after the fusion process is 13250* 5632. The k-fold cross-validation method is used for the validation of this multiclass classification.

### Experiments performed based on feature optimizer and no. of folds

To perform the experiments two different feature optimizers are used. First Dragonfly feature optimizer is selected for the selection of optimal features second is the GA feature optimizer to perform the experiments. The parameters selected for the Dragonfly feature optimizer are discussed in Table 5. A total of 4 experiments were executed based on Dragonfly optimization; two experiments were executed with 5 folds and 2 experiments were executed with 10 folds cross-validation. A different number of validations is selected to check out the difference if it exists by changing the number of folds for validation purposes.

**Table 5:** Parameters Values used for Dragonfly optimization.

Parameters Names	Values
Maximum iteration	25
No of Dragonflies	10
No. of folds	5, 10
No. of selected features	950, 1250
Deep CNN Networks	Proposed (Up-to-the-minute-Net)
	Inception-Resnet-V2

Two experiments are performed with 950 selected features with 5- and 10-fold cross-validation. Two experiments are performed with 1250 selected features on both folds for validation. In this manuscript, the model is also tested with a GA feature optimizer to attain the best results. In the following category total of 4 experiments are performed based on GA features optimizer. 2 experiments are performed with 5 folds and two are performed with 10 folds. Parameter values used for experiments are elaborated in Table 6.

**Table 6:** Parameters values used for GA optimization.

Parameter’s name	Values
Maximum iteration	25
No of chromosomes	10
Crossover Rate	0.6
Mutation Rates	0.001
No. of folds	5, 10
No. of selected features	500, 2500
Deep CNN Networks	Proposed (up-to-the-minute-Net), Inception-Resnet-V2

Two experiments are performed with 500 selected features with 5- and 10-fold cross-validation two experiments are performed with 2500 on both folds for validation. The outcomes of all 8 experiments are discussed in Table 7 which are comprised of Pre, Rec, F1-Sc, and Accuracy.

**Table 7:** Performance evaluation of all experiments.

Experiments	Selected No. of Features	Feature Optimizer	Cross Fold Validation	Classifier	Pre	Rec	F1-Sc	Accuracy
1	950	Dragonfly	5	Linear-SVM Quad-SVM **Cu-SVM** FineG-SVM MedG-SVM CoarG-SVM	0.96 0.98 **0.99** 0.80 0.99 0.81	0.95 0.98 **0.99** 0.80 0.99 0.80	0.96 0.98 **0.99** 0.81 0.99 0.80	95.8% 98.9% **99.3%** 79.8% 99.2% 90.8%
2			10	Linear-SVM Quad-SVM Cu-SVM FineG-SVM **MedG-SVM** CoarG-SVM	0.96 0.99 0.99 0.80 **0.99** 0.91	0.96 0.99 0.99 0.80 **0.99** 0.91	0.96 0.99 0.99 0.81 **0.99** 0.91	96.1% 99.0% 99.0% 79.8% **99.3%** 90.8%
3	1250		5	Linear-SVM Quad-SVM **Cu-SVM** FineG-SVM MedG-SVM CoarG-SVM	0.99 1.00 **1.00** 0.76 1.00 0.98	0.99 1.00 **1.00** 0.89 1.00 0.97	0.99 1.00 **1.00** 0.79 1.00 0.97	98.8% 98.9% **99.2%** 80.1% 99.0% 90.3%
4			10	Linear-SVM Quad-SVM **Cu-SVM** FineG-SVM MedG-SVM CoarG-SVM	0.97 0.99 **0.99** 0.80 0.99 0.91	0.97 0.99 **0.99** 0.80 0.99 0.91	0.97 0.99 **0.99** 0.80 0.99 0.91	97.0% 99.0% **99.3%** 81.0% 99.2% 91.0%
5	500	GA	5	Linear-SVM Quad-SVM **Cu-SVM** FineG-SVM MedG-SVM CoarG-SVM	0.94 0.99 **0.99** 0.79 0.99 0.89	0.94 0.99 **0.99** 0.90 0.99 0.89	0.94 0.99 **0.99** 0.81 0.99 0.89	95.2% 98.7% **99.2%** 79.2% 99.0% 89.1%
6			10	Linear-SVM Quad-SVM **Cu-SVM** FineG-SVM MedG-SVM CoarG-SVM	0.95 0.98 **0.99** 0.80 0.99 0.89	0.95 0.99 **0.99** 0.90 0.98 0.90	0.95 0.98 **0.99** 0.83 0.99 0.90	94.6% 99.0% **99.2%** 81.4% 99.2% 89.6%
7	2500		5	Linear-SVM Quad-SVM **Cu-SVM** FineG-SVM MedG-SVM CoarG-SVM	1.00 1.00 **1.00** 0.76 1.00 0.97	1.00 1.00 **1.00** 0.90 1.00 0.97	1.00 1.00 **1.00** 0.78 1.00 0.97	99.5% 99.8% **99.9%** 76.8% 99.8% 96.6%
8			10	Linear-SVM **Quad-SVM** **Cu-SVM** FineG-SVM **MedG-SVM** CoarG-SVM	0.99 **1.00** **1.00** 0.78 **1.00** 0.97	0.99 **1.00** **1.00** 0.90 **1.00** 0.97	0.99 **1.00** **1.00** 0.77 **1.00** 0.97	99.5% **99.8%** **99.8%** 78.9% **99.8%** 97.0%

**Fig 4:**
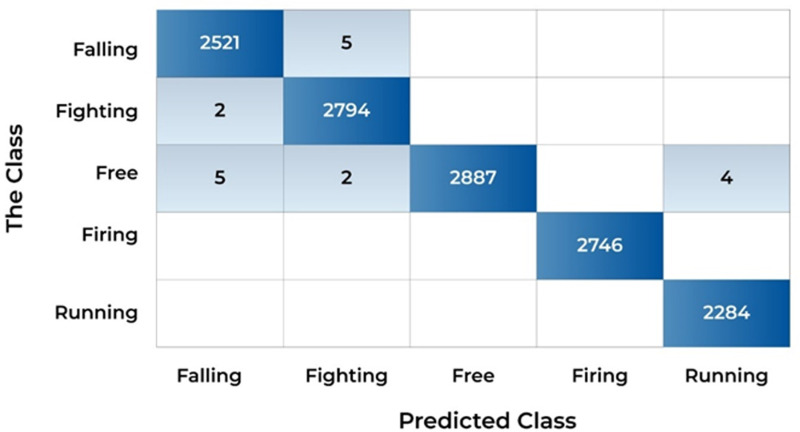
CM of highest results obtained.

The highest accuracy achieved is 99.9% with a Pre of 1.00, Rec of 1.00, and F1-Sc of 1.00. In this experiment, the selected number of optimal features is 2500 from total features of 5632. The CM in (Fig 4) shows how well the model classified the different anomaly classes in the SAR dataset and it presents the CM of the experiment with the highest accuracy. The CM details the performance of the model in classifying different anomaly types, with metrics such as TP, FP, TN, and FN.

**Fig 5:**
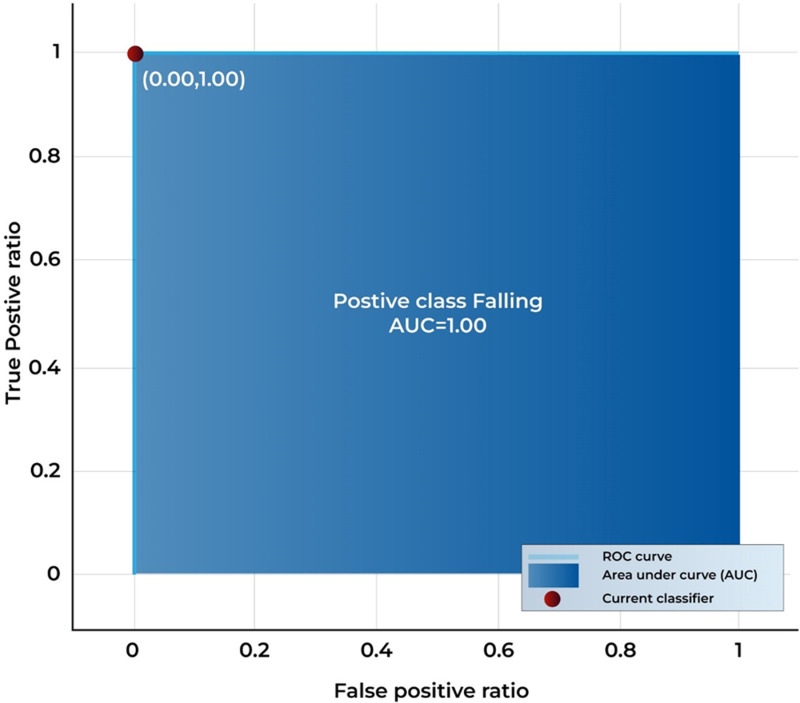
ROC of highest results obtained.

The ROC curve in (Fig 5) visualizes the trade-off between TP rates (sensitivity) and FP rates across different classification thresholds. The AUC value close to 1.0 signifies that the model is highly effective at distinguishing between anomaly and non-anomaly cases. The curve in this Fig shows that the model’s predictive power is excellent, as it achieves near-perfect classification with minimal FP and FN.

**Fig 6:**
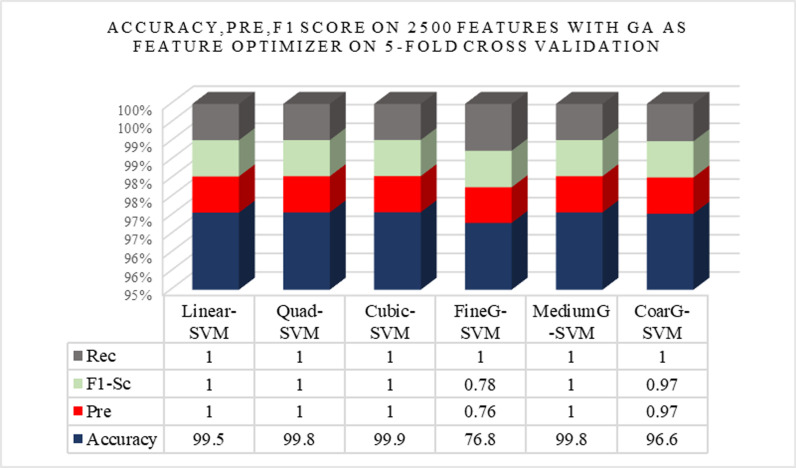
Graphical representation of Accuracy, Pre, Rec, and F1 Score on 2500 features with GA optimization on 5-fold cross-validation.

The (Fig 6) shows the performance metrics for Experiment 7, which achieved the highest accuracy. The fig includes graphical representations of Accuracy, Pre, Rec, and F1-Sc for the model trained with 2500 features using GA optimization and 5-fold cross-validation. This fig emphasizes the model’s superior performance across all metrics, with perfect scores indicating that the model is highly effective at detecting anomalies. The detailed performance metrics underscore the impact of optimal feature selection and the effectiveness of the GA optimization technique.

No. of the folds on which the highest accuracy is achieved is 5. In this experiment, the data is trained on the testing and training ratio of 20:80. The specified portion of testing and training data is changed after every iteration.

### Accuracy over selected features

The selection of optimal features has a great impact on accuracy. A total 4 number of features are selected for experimentation those are 500,950,1250,2500. Table 8 describes the association between the features and accuracy.

**Table 8:** Accuracy over selected features.

Sr. No	No. of features	Accuracy
1	500	99.2%
2	950	99.3%
3	1250	99.3%
4	2500	99.9%

**Fig 7:**
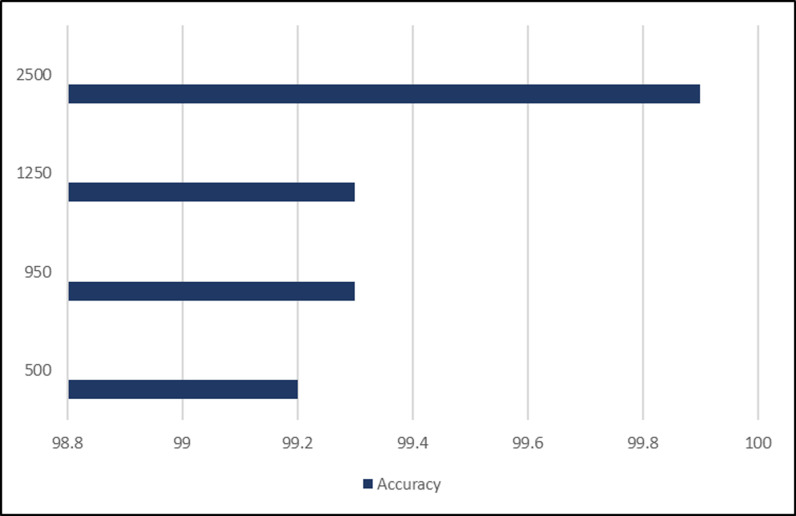
The relational graph between No. of features and Accuracy.

The selection of these optimal features is random, as the total fused feature is 5632 in number. The careful selection of features shown in (Fig 7), rather than simply using all available features, leads to significant improvements in accuracy. The performance improvements as the number of features increases show that the model can leverage additional information up to a point, after which further increases offer limited benefits.

The highest accuracy achieved is 99.9% on 2500 features on Cu-SVM with GA optimization on 5-fold cross-validation.

### Comparison with existing results

This section compares the previously published work of [69]. Heretofore the number of experiments done was 5. The number of selected features was 100, 250, 500, 450, 1000. The number of folds selected for all experiments was 5 folds. ASO is used for feature optimization of all experiments. The highest result achieved was 99.3% on the Cu-SVM of a selected number of 500 features. Table 9 provides a widespread evaluation of the proposed work with prevailing work.

**Table 9:** A widespread comparison of proposed work with existing work.

Explorations	Year	No. of experiments	Feature Optimizer	No. of Folds	No. of Selected Features	Accuracy
Existing Work[69]	2021	1	ASO	5	100	98.4%
		2			250	98.9%
		3			500	99.3%
		4			750	99.1%
		5			1000	99.2%
Proposed	2022	1	Dragonfly	5	950	99.3%
		2		10		99.3%
		3		5	1250	99.2%
		4		10		99.3%
		5	GA	5	500	99.2%
		6		10		99.2%
		7		5	2500	99.9%
		8		10		99.8%

In the proposed work a total of 8 experiments are executed with the selected features of 500,950,1250 and 2500. The experiments are accomplished, and results are obtained by the two feature optimizers the Dragonfly algorithm and GA. No of the folds selected are 5 folds and 10 folds for cross-validation. The highest accuracy acquired is 99.9% from 2500 selected features on Cu-SVM with 5-fold cross-validation. While [69] achieved 99.3% accuracy with 500 features using the entropy-coded ant colony optimization and L4-branched-ActionNet, our model achieved the same accuracy with 950 features using the Up-to-the-Minute-Net and GA optimizer. However, our model was designed to explore the impact of using a larger feature set (up to 2500 features), ultimately achieving 99.9% accuracy. This suggests that while [69] Maybe more efficiently with fewer features, our approach excels when higher feature counts yield better performance. Future work will focus on optimizing feature selection to balance accuracy and computational efficiency for real-world applications.

### Statistical analysis

To ensure the robustness and reliability of our model, we employed rigorous statistical methods throughout the study. We utilized 5-fold and 10-fold cross-validation to validate the model’s efficiency, ensuring generalizability across different data splits. Additionally, we implemented two distinct feature optimization techniques—Dragonfly and GA—to select the most relevant features, testing different feature subsets to optimize accuracy and reliability. The models were evaluated using key performance metrics such as Pre, Rec, F1-Sc, and accuracy, across multiple iterations and parameter settings, confirming the consistency and validity of our findings. These comprehensive steps ensure that the statistical analysis conducted is thorough and methodologically sound.

The base paper achieved a prediction speed of 650 obs/sec, while the proposed model achieved 87 obs/sec. This speed discrepancy can largely be attributed to the computational power available in the respective environments. The base paper benefited from the use of a dedicated NVIDIA GTX 1070 GPU, which is designed to handle intensive computational tasks such as model training and prediction at a much faster rate than a CPU. In contrast, the proposed model was run on a CPU-based system without GPU acceleration, which naturally results in slower processing speeds. Although the prediction speed of the proposed model is lower, the focus of our research is on improving accuracy, achieving 99.9%, as compared to the 99.3% accuracy reported in [69]. The increase in accuracy, despite the computational limitations, demonstrates the effectiveness of our feature extraction and selection approach, making the trade-off between speed and accuracy a key consideration. For applications where high accuracy is more critical than prediction speed, the proposed model offers a valuable alternative.

In on our feature selection process and the trends observed with the use of 500, 950,1250, and 2500 features, we anticipate a marginal increase in accuracy beyond 99.9% when using the full feature set. The primary goal of our feature selection was to find an optimal balance between computational efficiency and accuracy, as the marginal increase in accuracy with significantly more features is unlikely to justify the additional computational cost. In real-time applications where speed is crucial, a model with fewer features might be preferable, whereas in other cases, the higher accuracy of the proposed model may justify the additional computational time.

## Discussion and limitations

The importance of anomaly recognition in surveillance has been discussed before. For this purpose, a model is designed and tested with different experiments. This subdivision mounts the experiments and results of the proposed approaches and the relative comparison to the existing techniques. The experiments are conducted with the standard benchmark dataset Suspicious Activity Recognition (SAR). In the paper, a list of nomenclature is presented in Table 1 and a comparative analysis of existing techniques to detect anomalies in surveillance with the help of DL is presented in Table 2. Fig 1 shows the flow chart of the proposed approach. Table 3 shows the detailed layer’s configuration of the proposed Up-to-the-Minute-Net. Fig 2 depicts the detailed layered architecture of the proposed DCNN net. Table 4 shows the hyperparameters and their values for the proposed Up-to-the-Minute-net. The augmented dataset is used for experimentation purposes and a graphic illustration of the dataset is shown in Fig 3. The extracted features from both DNNs got fused which are passed to two feature optimizers to attain the best results. The hyperparameters set for the experimentation based on the Dragonfly features optimizer are discussed in Table 5. The hyperparameters set for the experimentation based on the GA features optimizer are discussed in Table 6. The selected features were passed to two validation methods, 5- and 10-fold cross-validation methods. The outcome of all 8 experiments is discussed in Table 7 comprised of Pre, Rec, F1-Sc, and Accuracy. The highest results were achieved in experiment 7 on Cu-SVM on 5-fold cross-validation on 2500 features with GA features optimizer. The CM and ROC of the highest accuracy are represented in Fig 4. The ROC of the highest obtained is shown in Fig 5. The Pre, Rec, F1-Sc, and accuracy of experiment 7 are represented graphically in Fig 6. The selection of several features has a great impact on results. The highest accuracy obtained on selected features is discussed in Table 8 and it is graphically depicted in Fig 7. For evaluation purposes, a comprehensive comparison of the proposed work with existing work is discussed in Table 9.

While our proposed framework demonstrates significant improvements in anomaly recognition accuracy, there are several limitations to consider. The method’s performance may vary when applied to larger or more diverse datasets outside of the benchmark dataset used in this study. Additionally, the computational complexity of the DL models and optimization algorithms may pose challenges in real-time applications or on resource-constrained devices. Further research is needed to evaluate the scalability and robustness of our approach in different operational environments and to address these practical constraints.

## Conclusion and future work

Within this manuscript, a DCNN-branched model named Up-to-the-Minute-Net is anticipated. The benchmark dataset SAR is used for experiments. The dataset consists of 5 classes of anomalies. After the preprocessing of images, the features are extracted from the proposed DCNN. Another pre-trained deep neural network Inception-Resnet-V2 is used for feature extraction. The extracted features from both DCNNs were fused. In this research we proposed two methodologies belonging to optimization, the experiments are performed on the fused features by two different feature optimizers for the selection of optimal features. The feature selection algorithms Dragonfly and GA are used for the optimization problem. The results are taken on 5 and 10 folds as well to pattern the variance. The total number of experiments performed is 8. Four experiments are performed by the Dragonfly feature optimizer and four are performed by the GA features optimizer. The number of features selected for different experiments are 500,950,1250 and 2500, The highest accuracy is 99.9% achieved on Cu-SVM on selected 2500 features on 5-fold cross-validation based on GA optimization. While the GA optimizer with 2500 features provided the highest accuracy, it is important to consider the computational requirements associated with this feature set. In real-world applications, the increased number of features may result in higher computational overhead, affecting processing time and memory usage. A comparison between GA with 2500 features and GA with 500 features highlights this trade-off, where the model with 500 features, although slightly lower in accuracy (99.2%), may be more suitable for resource-constrained environments. Future work will focus on evaluating these computational aspects to optimize the framework for practical scenarios.
